# Memory mechanisms for behavioural change in Bayesian individual-level spatial epidemic models

**DOI:** 10.1016/j.idm.2026.05.008

**Published:** 2026-06-02

**Authors:** Yicheng Mao, Rob Deardon, Lorna E. Deeth

**Affiliations:** a Department of Data Analytics and Digitalization, Maastricht University, P.O. Box 616, 6200 MD, Maastricht, the Netherlands; b Department of Mathematics and Statistics, University of Calgary, University Drive NW, Calgary, T2N 1N4, Canada; c Faculty of Veterinary Medicine, University of Calgary, University Drive NW, Calgary, T2N 1N4, Canada; d Department of Mathematics and Statistics, University of Guelph, 50 Stone Rd. E., Guelph, Ontario, N1G 2W1, Canada

**Keywords:** Behavioural change, Individual-level models, Infectious disease modelling, SIR models, Bayesian inference

## Abstract

Accurate modelling of infectious disease transmission often requires capturing how individuals adjust their behaviours in response to evolving epidemic conditions. While recently developed behavioural change epidemic models attempt to acknowledge such dynamics, the role of memory in shaping perceived risk has been treated in an ad hoc fashion. This study develops a data-driven framework of memory enhanced behavioural change individual-level models (MEBC-ILMs) that incorporate four distinct memory specifications: memoryless, sliding window, power-law decay, and exponential decay. These models allow behavioural responses to reflect varying assumptions about how past epidemiological information informs risk perception, with memory features inferred from epidemic data. Simulation experiments show that MEBC-ILMs can reliably recover key transmission and behavioural parameters under different memory settings and exhibit robust predictive performance even when the assumed memory structure differs from the true process. In contrast, the basic BC-ILM can perform poorly when memory affects present behaviour but is not accounted for in the model. Applying our framework to data from the 2001 U.K. foot and mouth disease epidemic illustrates how it can represent behavioural effects and explore plausible memory structures when fitted to real-world data.

## 1 Introduction

The devastating impact of epidemics underscores the urgent need for robust tools to understand, predict, and mitigate disease spread. Mathematical epidemic models have proven invaluable for this purpose, offering critical insights into transmission dynamics and guiding public health interventions. However, disease propagation is not solely a biological phenomenon; it is intricately linked with and profoundly shaped by population behaviours and their dynamics. For example, during COVID-19, adjustments in travel patterns, social interactions, and mask-wearing practices can help slow the spread of the disease and reduce the overall scale of the outbreak (Mendez-Brito et al., 2021; Wang et al., 2021). Similarly, changes in livestock management have been critical for controlling animal epidemics such as foot-and-mouth disease (FMD), including measures like restrictions on animal movement, mass culling, and vaccination programs (Pomeroy et al., 2017).

Building on the recognition of human behaviour's critical role, an increasing body of research has sought to integrate behavioural factors into epidemic models to provide a more accurate representation of disease spread (Weitz et al., 2020). Early efforts in this area were systematically reviewed by Funk et al. (2010), who highlighted that most models rely on disease prevalence as the key information source driving behavioural change and underscored the ongoing challenge of parameterizing how individuals act on such information. An updated review by Verelst et al. (2016) further pointed out that the vast majority of studies have focused solely on model specification and simulation-based analysis, with limited efforts to validate assumptions using real-world data. More recently, Ward et al., 2023, Ward et al., 2025 proposed a stochastic Bayesian framework that integrates behavioural change into data-driven compartmental epidemic models through an “alarm” function. This function quantifies public risk perception and modulates individual susceptibility accordingly, and has been validated using real-world data.

Despite these advances, a significant gap persists in how behavioural change is typically modeled. Many existing approaches either base individual responses solely on current information, without explicitly considering accumulated historical knowledge, or rely on ad hoc assumptions about how memory shapes behaviour, thereby imposing a rigid evolution of responses. These approaches rarely allow the data to identify which historical signals matter and over what time horizon they influence decisions, which can lead to misjudging the timing or magnitude of responses such as risk fatigue or sustained vigilance (d’Onofrio & Manfredi, 2009; Rypdal et al., 2020). To address these limitations, we develop a data-driven ILM framework that endogenizes behavioural change within the epidemic process and the memory mechanism that drives it. The model links individual susceptibility to recent epidemic history and learns from the data whether memory is present and what form it takes.

Epidemic modelling approaches span a spectrum of granularity, from aggregated population-level models to highly detailed agent-based simulations (Ajelli et al., 2010). While population-averaged frameworks are computationally efficient and effective for broad trends in homogeneous groups, they often simplify complex individual behaviours and local interactions. At the other end of the spectrum, agent-based or metapopulation models can capture intricate individual dynamics and spatial patterns (Meloni et al., 2011), but their extensive data requirements can pose significant challenges for empirical application. This study therefore adopts the individual-level model (ILM) framework, following Deardon et al. (2010), which offers a practical balance between detailed representation and tractability. Building on this foundation and related work that incorporates behavioural change within spatial ILMs (Ward et al., 2025), we introduce memory mechanisms into this framework. Specifically, we propose a class of Memory-Enhanced Behavioural Change Individual-Level Models (MEBC-ILMs), each integrating a distinct memory mechanism into individual decision-making. These new MEBC-ILMs remain flexible to available data and are well suited to studying how memory shapes behavioural responses in a spatially explicit setting. The key aim is to learn from epidemic data how these mechanisms select and incorporate historical signals, thereby capturing the dynamic and adaptive nature of behavioural responses and improving the realism and predictive performance of epidemic forecasts.

The remainder of this paper is organized as follows. Section 2 details the design of our proposed MEBC-ILM framework. Section 3 investigates the properties of these models through extensive simulations. Section 4 further examines the performance of these models under conditions of model misspecification via simulation. Section 5 applies our models to the 2001 U.K. FMD outbreak data. Finally, Section 6 concludes with a summary of our findings and outlines avenues for future research.

## 2 Methods

### 2.1 Spatial individual-level models

Deardon et al. (2010) proposed an ILM framework in which infection probabilities are defined over discrete time intervals using data collected at the individual level. In spatial ILMs, each individual is treated as a distinct unit in both spatial and temporal dimensions, with events assigned to time step *t* interpreted as having occurred at some point within the continuous interval [*t*, *t* + 1).

Spatial ILMs are typically implemented within compartmental frameworks such as the SIR model (Kermack & McKendrick, 1927). In this framework, the population is divided into three mutually exclusive compartments based on disease status: susceptible (*S*), infectious (*I*), and removed (*R*). Individuals transition sequentially from being susceptible to becoming infectious, and ultimately to a removed state in which they can neither transmit nor contract the disease again. At any discrete time point, each individual belongs to exactly one of these compartments, and transitions are permitted only in a forward direction. The transition from susceptible to infectious is governed by a probability of infection, while the shift from infectious to removed depends on the infectious period. This infectious period may be assumed to vary across individuals or remain constant, and may be treated as either known or subject to estimation. In this work, for simplicity we assume a fixed and known infectious period that is identical for all individuals.

Deardon et al. (2010) presented the general form of an epidemic ILM as follows:

(1) $$P\left(i,t\right)=1-\exp\left[-\Omega_{S}\left(i\right)\underset{j\in I\left(t\right)}{\sum }\Omega_{T}\left(j\right)\kappa \left(i,j\right)+\epsilon \left(i,t\right)\right],$$

where *P*(*i*, *t*) represents the probability that a susceptible individual *i* becomes infected at time *t* and subsequently enters the infectious state at time *t* + 1. Here, *I*(*t*) denotes the set of infectious individuals at time *t*. The term Ω_*S*_(*i*) captures susceptibility-related factors that influence the likelihood of individual *i* acquiring infection. Similarly, Ω_*T*_(*j*) reflects transmissibility-related characteristics of infectious individual *j*, determining their potential to transmit the disease to a susceptible contact. The infection kernel *κ*(*i*, *j*) accounts for contextual elements such as spatial distance between individuals *i* and *j*, shaping the intensity of transmission between them. The spark term *ϵ*(*i*, *t*) encompasses additional sources of infection not explicitly captured by the model's structure.

In this study, we use a typical form of a spatial ILM:

(2) $$P\left(i,t\right)=1-\exp\left[-\alpha \underset{j\in I\left(t\right)}{\sum }d_{ij}^{-\beta }\right].$$

In this model specification, we assume homogeneous susceptibility and transmissibility across all individuals, with Ω_*S*_(*i*) = *α* > 0 and Ω_*T*_(*j*) = 1. The infection kernel follows a distance-based power-law form, where *d*_*ij*_ denotes the Euclidean distance between susceptible individual *i* and infectious individual *j*. The spatial parameter *β* controls how quickly transmission risk decreases with increasing distance. For simplicity, the spark term *ϵ*(*i*, *t*) is set to zero.

### 2.2 Behavioural change spatial individual-level models

Building on the ILM framework, Ward et al. (2025) proposed a series of BC-ILMs. These models are characterized by the inclusion of an alarm function *a*_*t*_, which quantifies the population's perceived risk of infection at time *t*. A typical form of BC-ILM integrates *a*_*t*_ into the infection probability expression by adjusting individual susceptibility as follows:

(3) $$P\left(i,t\right)=1-\exp\left[-\alpha \left(1-a_{t}\right)\underset{j\in I\left(t\right)}{\sum }d_{ij}^{-\beta }\right],\;0\leq a_{t}\leq 1.$$

In this formulation, *a*_*t*_ serves as a multiplicative modifier of susceptibility, with higher values of *a*_*t*_ reflecting stronger behavioural responses that lower transmission risk, such as increased mask usage or reduced travel. This is not the only possible way to incorporate behavioural change into the infection model. In Ward et al. (2025), behavioural responses may also be introduced through alternative components of the transmission mechanism, including reformulations of the infection kernel term. In the present study, we focus on the susceptibility-based scaling formulation for the main analysis, while additional discussions under alternative behavioural formulations are provided in the Supplementary Material.

Ward et al. (2025) proposed several alternative formulations for the alarm function. In this study, we focus on the exponential alarm function, which can be written as:

(4) $$a_{t}=1-\exp\left(-\delta \;\psi_{t}\right),$$

where 0 < *δ* < 1 is a positive scaling parameter. Notably, setting *δ* = 0 yields *a*_*t*_ = 0 for all *t*, in which case the BC-ILM collapses to the original ILM. *ψ*_*t*_ denotes the effective perceived risk signal at time *t*, derived from available epidemiological indicators, such as the time series of disease prevalence. In Ward et al. (2025), the construction of *ψ*_*t*_ is based on a highly simplified assumption that individuals form their perception of risk solely based on the previous time point's prevalence. The temporal window over which prevalence data are integrated, as well as the relative importance assigned to each time point's value in shaping perceived risk, are not explicitly modeled or discussed. In this study, we address this limitation by introducing a memory-based mechanism that allows the risk signal to be constructed as a function of past epidemiological data, with the contribution of earlier observations determined by the memory model and estimated from the data.

### 2.3 Memory enhanced behavioural change spatial individual-level models

In this study, we propose four alternative memory mechanisms and incorporate them into the BC-ILM framework. We begin by embedding the baseline BC-ILM proposed by Ward et al. (2025) into our unified framework of MEBC-ILMs. This formulation assumes that individuals respond only to the most recently observed prevalence, without retaining or considering any earlier information. The corresponding perceived risk signal *ψ*_*t*_ is defined as:

(5) $$\psi_{t}=h_{t-1},$$

where *h*_*t*−1_ denotes the disease prevalence observed at time *t* − 1. We refer to this structure as the memoryless mechanism, as it assumes that individuals react exclusively to the latest available information while disregarding all earlier observations. In other words, the formation of risk perception is based solely on the previous time point's prevalence, implying that earlier data are either forgotten or considered irrelevant in shaping current behavioural responses.

The second memory structure assumes that individuals form their risk perception based on the average prevalence over a fixed number of previous time steps, denoted by *k*_max_. This represents a sliding window of memory, in which individuals assign equal importance to each time point's prevalence within the window while disregarding earlier information. The effective signal *ψ*_*t*_ is defined as:

(6) $$\psi_{t}=\frac{1}{k_{\text{max}}}\overset{k_{\text{max}}}{\underset{k=1}{\sum }}h_{t-k},$$

where *h*_*t*−*k*_ denotes the prevalence at time *t* − *k*, and *k*_max_ determines the length of the memory window. In our implementation, *k*_max_ is treated as an unknown discrete parameter and estimated from the data, allowing the model to infer the effective span of recent information. When *k*_max_ = 1, this specification reduces to the memoryless model. We refer to this mechanism as the sliding window memory model. This specification provides a simple representation of bounded memory, in which only recent prevalence values contribute to the perceived risk signal.

The third memory structure assumes that individuals integrate past prevalence values with decreasing sensitivity over time, following a power-law decay function. The perceived risk signal *ψ*_*t*_ is defined as:

(7) $$\psi_{t}=\overset{t-1}{\underset{k=1}{\sum }}k^{-\lambda_{P}}h_{t-k},$$

where *λ*_*P*_ > 0 is the decay parameter controlling how quickly the influence of past prevalence declines with the lag. We refer to this mechanism as the power-law decay memory model. This formulation is consistent with theories of human memory suggesting that the influence of past information often decays gradually and retains a relatively long tail, so that earlier events are not abruptly forgotten but instead continue to exert diminishing influence over time (Rubin & Wenzel, 1996; Wixted & Ebbesen, 1991).

Similarly, we introduce an exponential decay memory model, in which past prevalence values are discounted exponentially over time:

(8) $$\psi_{t}=\overset{t-1}{\underset{k=1}{\sum }}e^{-\lambda_{E}\left(k-1\right)}h_{t-k},$$

where *λ*_*E*_ > 0 governs the rate at which historical information is discounted. We refer to this mechanism as the exponential decay memory model. Compared with the power-law formulation, this mechanism places relatively greater emphasis on recent prevalence and implies that the influence of more distant observations declines more rapidly. Such a formulation provides a simple representation of memory processes in which recent information dominates current perception and older information fades quickly.

For these decay-based memory models, it is useful to characterize how the contribution of past prevalence declines with the lag. We therefore define the *ℓ*-day decay level, denoted by *D*_*ℓ*_, as the contribution of prevalence observed *ℓ* time steps in the past relative to that of the most recent observation. Under the power-law decay model,

(9) $$D_{\ell }=\ell^{-\lambda_{P}},$$

whereas under the exponential decay model,

(10) $$D_{\ell }=e^{-\lambda_{E}\left(\ell -1\right)}.$$

In both cases, *D*_1_ = 1 by construction. For a fixed lag *ℓ*, larger values of *D*_*ℓ*_ indicate that information from *ℓ* time steps earlier retains a greater contribution to the perceived risk signal, whereas smaller values indicate faster decay of past information by that lag. This quantity provides a simple and interpretable way to compare how much information from the same lag is retained under different memory decay formulations.

By substituting Eq. (5) to Eq. (8) into the general behavioural formulation in Eq. (4), and integrating the resulting *a*_*t*_ expressions into the infection probability in Eq. (3), we obtain four distinct MEBC-ILMs, each corresponding to a different assumption about how individuals form perceived risk over time.

### 2.4 Bayesian inference

Inference is conducted within a Bayesian framework. Consistent with epidemic models built within the SIR framework, the spatial MEBC-ILM defines the likelihood at each time point based on the observed infection states of individuals. Specifically, at time *t*, the conditional likelihood is defined as the product of the infection probabilities for individuals who become newly infectious at *t* + 1, and the probabilities of remaining uninfected for those who continue to be susceptible at *t* + 1. Let *S*(*t*), *I*(*t*), and *R*(*t*) denote the sets of susceptible, infectious, and removed individuals at time *t*, respectively. The likelihood can then be expressed as:

(11) $$f_{t}\left(S\left(t\right),I\left(t\right),R\left(t\right)|\Theta \right)=\left[\underset{i\in I\left(t+1\right)\setminus I\left(t\right)}{\prod }P\left(i,t\right)\right]\left[\underset{i\in S\left(t+1\right)}{\prod }\left(1-P\left(i,t\right)\right)\right],$$

where Θ denotes the set of model parameters. The full likelihood of the epidemic data *D*, observed over all time points in the study period, is obtained by taking the product of the conditional likelihoods at each time step:

(12) $$f\left(D|\Theta \right)=\overset{t_{\text{max}}-1}{\underset{t=1}{\prod }}f_{t}\left(S\left(t\right),I\left(t\right),R\left(t\right)|\Theta \right).$$

Placing a prior *p*(Θ) on the parameters yields the posterior *π*(Θ∣*D*) ∝ *f*(*D*∣Θ) *p*(Θ). We employ Markov chain Monte Carlo (MCMC) for parameter estimation. Specifically, posterior samples are obtained using an adaptive random walk Metropolis–Hastings sampler (Roberts & Rosenthal, 2009). All computations were implemented in Julia v1.11.5.

## 3 Simulation study

In this section, we consider simulation experiments to evaluate the performance of our MEBC-ILMs under various settings. Specifically, we examine the accuracy of parameter estimation and predictive performance across different memory structures. Section 3.1 outlines the simulation design, while Section 3.2 presents a detailed analysis of the results.

### 3.1 Simulation setup

Our simulation study focuses on three distinct memory mechanisms: the sliding window, power-law decay, and exponential decay models. For each mechanism, we generate scenarios representing both “short” and “long” memory by varying the corresponding memory-related parameters. It is important to note that the labels “short” and “long” are used in a relative sense, denoting comparative differences within each mechanism rather than absolute durations.

**Fig. 1:**
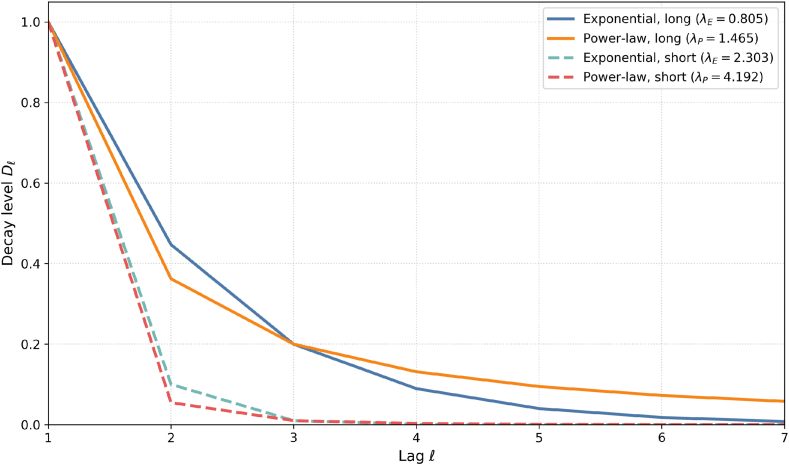
Values of *D*_*ℓ*_ over a 7-day period for the power-law and exponential memory models.

Table 1 presents the specific simulation settings for all six scenarios. The values for the susceptibility parameter *α*, the spatial parameter *β*, and the scale parameter *δ* are chosen in accordance with the configurations used in Ward et al. (2025). For the power-law and exponential decay models, the values of *λ*_*P*_ and *λ*_*E*_ were selected using the 3-day decay level *D*_3_ introduced in Section 2.3. Under the short-memory condition, parameters were chosen such that *D*_3_ ≈ 0.01, meaning that prevalence observed three time steps earlier retains approximately 1% of the contribution of the most recent observation. Under the long-memory condition, parameters were chosen such that *D*_3_ ≈ 0.20, indicating a substantially slower decay. Fig. 1 shows the corresponding values of *D*_*ℓ*_ over a 7-day period.

**Table 1:** Simulation settings under different memory mechanisms.

Memory mechanism	*α*	*β*	*δ*	Memory parameter	Memory length
Sliding window	2.4	2	0.01	*k*_max_ = 7	Long
Sliding window	2.4	2	0.01	*k*_max_ = 3	Short
Power-law	2.4	2	0.01	*λ*_*P*_ = 1.465	Long
Power-law	2.4	2	0.01	*λ*_*P*_ = 4.192	Short
Exponential	2.4	2	0.01	*λ*_*E*_ = 0.805	Long
Exponential	2.4	2	0.01	*λ*_*E*_ = 2.303	Short

For the sliding window model, an analogous decay profile is not available, since all prevalence values within the window contribute equally and all earlier values contribute nothing. We therefore consider two commonly used window lengths, *k*_max_ = 3 and *k*_max_ = 7, to represent shorter and longer bounded-memory settings.

For each memory specification, we simulated 20 independent populations, each comprising 1,000 individuals. The spatial locations of individuals were independently drawn from a uniform distribution over a 200 × 200 unit square, that is, x,y∼U(100,300), where U denotes the uniform distribution. Within each population, three individuals were randomly selected as the initial infectious cases. The infectious period was fixed at 3 days. Epidemic processes were then simulated under the SIR framework over a total of *t*_max_ = 30 discrete time steps.

For each scenario, three independent MCMC chains were run, each consisting of 50,000 iterations, with the initial 10,000 iterations discarded as burn-in. Convergence diagnostics were performed using the Gelman–Rubin–Brooks statistic (Brooks & Gelman, 1998; Gelman & Rubin, 1992). Vague or weakly informative priors were specified for all model parameters. Specifically, the susceptibility parameter *α* and the spatial transmission parameter *β* were both assigned uniform priors U(0,100). The scale parameter *δ* was given a Beta(1, 1) prior. For the sliding window memory model, the window size parameter *k*_max_ was assigned a discrete uniform prior $U_{d}\left(1,14\right)$. The decay rate parameters in the power-law and exponential memory models, *λ*_*P*_ and *λ*_*E*_, were both assigned Gamma(1, 1) priors.

To assess the models’ predictive accuracy, we examined the posterior predictive distribution (PPD) of the epidemic curve, defined as the number of new infections occurring at each time point during the epidemic period. Formally, the epidemic curve C is represented as:

(13) $$C={\left\{\left|I\left(t+1\right)\setminus I\left(t\right)\right|\right\}}_{t=1}^{t_{\text{max}}},$$

where *I*(*t*) denotes the set of infectious individuals at time *t*, and *t*_max_ is the final time point of the simulation period. The posterior predictive distribution of C was estimated by simulating epidemic trajectories from 500 posterior draws obtained via MCMC. For each time point, 95% highest posterior density intervals (HPDIs) were computed to evaluate how well the model captures the observed temporal dynamics of infection.

### 3.2 Simulation results

Convergence of all MCMC chains was confirmed using the Gelman–Rubin–Brooks diagnostic, with all potential scale reduction factors $\left(\hat{R}\right)$ below 1.05.

**Fig. 2:**
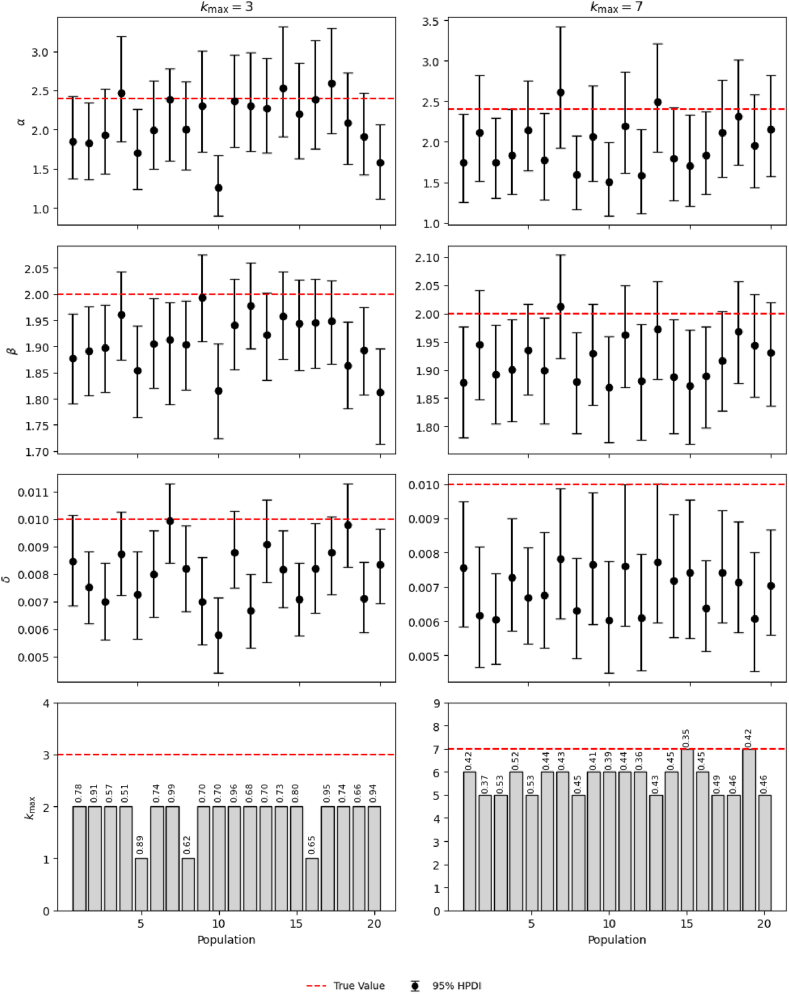
Posterior summaries of *α*, *β*, *δ*, and *k*_max_ under the sliding window memory specification.

Fig. 2 presents the posterior estimates of model parameters under the sliding window memory specification. For continuous parameters *α*, *β*, and *δ*, we report the posterior means and 95% HPDIs across 20 simulated populations. For the discrete memory parameter *k*_max_, we report the posterior mode along with its associated posterior probability in each case.

Consistent with findings reported in Ward et al. (2025), the posterior estimates of the key transmission-related parameters *α*, *β*, and the behavioural response parameter *δ* were consistently biased downward across all the simulated populations. These parameters jointly shape the epidemic growth rate in MEBC-ILMs, and the underestimation of one parameter can be partially compensated for by adjustments in the others. A lower value of *α* implies reduced susceptibility among individuals, thereby slowing epidemic spread. Similarly, a smaller *β* value leads to more spatially diffuse infections, as transmission is less constrained by distance. This not only accelerates the epidemic but also underplays spatial structure, a phenomenon commonly observed in spatial ILMs (Malik et al., 2016; Marion et al., 2022). Meanwhile, underestimating *δ* weakens the strength of the behavioural change effect, resulting in a faster-spreading epidemic as individuals respond less strongly to rising epidemic risk. Taken together, these patterns suggest that the model may underestimate the overall behavioural effect, and this is offset by downward adjustments in *α* and *β* to preserve epidemic dynamics.

A further explanation for this systematic underestimation may come from the discrete-time form of the infection likelihood. In the spatial ILM, infection at each time step is represented as a Bernoulli event for each susceptible individual, and the infection probability is determined by the combined contribution of all infectious individuals present at that time. Therefore, when several potential transmission events occur within the same local cluster and time interval, they are not represented separately in the likelihood, but instead enter through a single infection outcome. This aggregation can reduce the information available for distinguishing stronger from weaker transmission pressure, especially when several infectious individuals simultaneously contribute to the risk of infection. This loss of resolution may in turn be amplified in later epidemic stages, when susceptible depletion further limits the information available for disentangling transmission-related and behavioural parameters. To investigate this issue, we conducted a supplementary sensitivity analysis based on truncated observation windows, and the results are reported in the Supplementary Material.

The discrete memory parameter *k*_max_ also tended to be underestimated, with posterior modes falling below the true values in the majority of populations. This pattern suggests that the effective memory span inferred by the model is often shorter than the true behavioural horizon encoded in the data. Together with the downward bias in *α*, *β*, and *δ*, this result indicates that the posterior may favour weaker and shorter-lived behavioural responses when similar epidemic dynamics can already be reproduced through smaller values of the transmission-related and behaviour-related parameters.

**Fig. 3:**
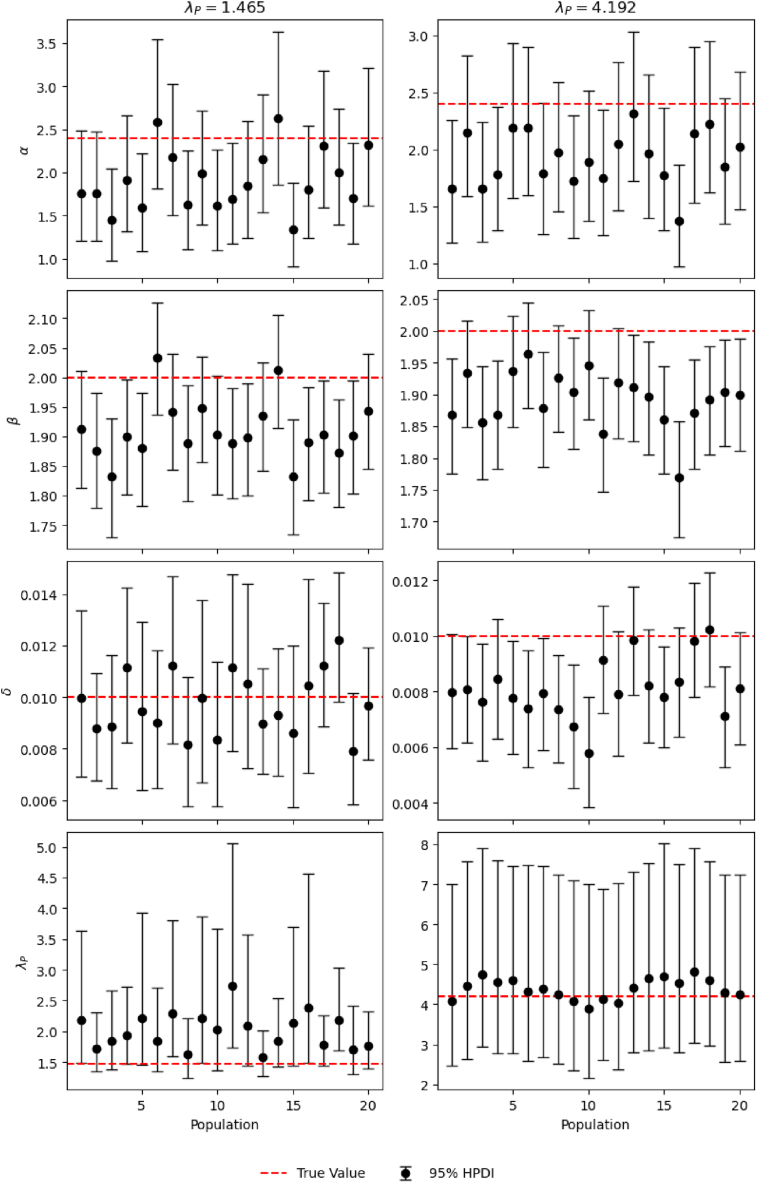
Posterior summaries of *α*, *β*, *δ*, and *λ*_*P*_ under the power-law memory specification.

Fig. 3 shows the posterior means and 95% HPDIs for model parameters under the power-law memory model with long (*λ*_*P*_ = 1.465) and short (*λ*_*P*_ = 4.192) memory. As with the sliding window memory, we again observe a general tendency toward underestimation for *α*, *β*, and *δ*, particularly under the shorter-memory setting. The estimation of the scale parameter *λ*_*P*_ appears more precise under the long memory condition, with posterior means closely aligning with the true value in most populations. In contrast, when the true memory decays more quickly, the model tends to underestimate *λ*_*P*_ substantially, with wide HPDIs indicating greater uncertainty.

The posterior estimates under the exponential memory model are similar to those from the power-law model. Detailed summaries are provided in the Supplementary Material. Across all three MEBC-ILMs, we observe a consistent underestimation of memory strength. This phenomenon may be attributed to the tendency of alarm functions with longer memory to incorporate additional historical prevalence data, which can dilute the behavioural signal or introduce noise. During MCMC sampling, such functions are more likely to yield poorer likelihood values, making the proposed parameters less consistent with observed epidemic dynamics and increasing their probability of rejection. Consequently, the posterior distribution may be biased toward shorter memory spans, even when longer memory better represents the true underlying behavioural process.

**Fig. 4:**
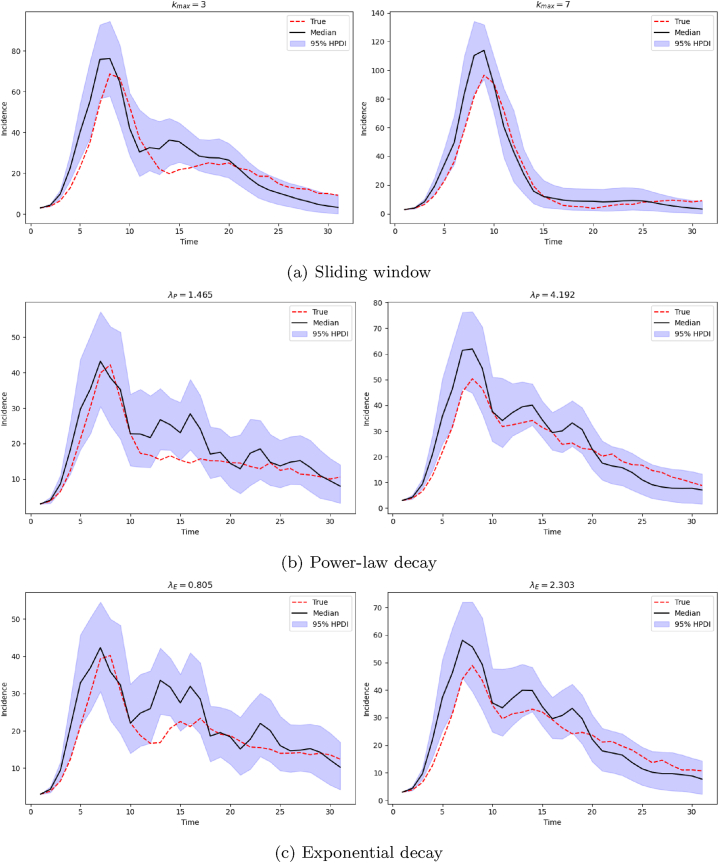
Comparison of incidence curves across three memory models (averaged over 20 simulated epidemics per model).

Fig. 4 displays the PPDs of the epidemic curves under all six scenarios, with each curve representing the average result across 20 simulated populations. In each panel, the red dashed line denotes the true epidemic curve, the black solid line indicates the posterior median, and the shaded blue region corresponds to the 95% HPDI. Overall, the predictive performance is satisfactory across all models and settings, with the true epidemic curve largely falling within the 95% HPDIs. For the sliding window model, predictive accuracy improves notably when the memory window is longer, as evidenced by the narrower HPDIs and closer alignment with the true curve. For both the power-law and exponential memory models, predictive performance is generally better under short-memory settings. In these cases, the 95% HPDIs closely track the true epidemic curve, capturing key inflection points with high precision. When the forgetting rate is lower, predictive accuracy slightly deteriorates, reflected in wider HPDIs and less precise tracking of the true trajectory. Nevertheless, the predictions remain within an acceptable range of uncertainty.

Given the interdependence among key model parameters, recovering accurate estimates within MEBC-ILMs remains inherently challenging. Although the posterior estimates obtained in our simulation study generally yield satisfactory predictive performance, systematic biases remain, particularly in behavioural and memory-related parameters. This suggests that individual parameter estimates should be interpreted with caution. These findings indicate that estimates in MEBC-ILMs should be evaluated in light of model complexity, the potential trade-offs among parameters during the inference process, and the effect of discrete-time aggregation in the likelihood formulation.

## 4 Memory mechanism misspecification

In this section, we use simulation experiments to examine the performance of the proposed MEBC-ILMs under model misspecification. Investigating model misspecification is important because, in practice, the true underlying behavioural process is often unknown and may differ from the functional form assumed in the model. Assessing the robustness of MEBC-ILMs to incorrect memory specifications provides insight into their reliability and the interpretability of parameter estimates in applied contexts.

Our simulation study considered all four memory mechanisms introduced in Section 2.3. We simulated 20 population datasets under each MEBC-ILM in turn. The true susceptibility parameter was set to *α* = 2.4, the spatial parameter to *β* = 2, and the scale parameter to *δ* = 0.01. The true memory parameters for the models were set as follows: *k*_max_ = 7 for the sliding window model, *λ*_*P*_ = 1.465 for the power-law model, and *λ*_*E*_ = 0.805 for the exponential model. Each population dataset was generated following the same procedure as described in Section 3.1. For each set of true datasets, we fit all four MEBC-ILM variants and evaluated predictive performance using the PPDs of the epidemic curve.

To complement the visual comparison of PPDs, we also used two quantitative criteria to evaluate predictive performance under misspecification. First, we computed the WAIC to assess overall model fit (Watanabe & Opper, 2010). Second, we computed the mean absolute error (MAE) between the averaged posterior median epidemic curve and the averaged true epidemic curve. Specifically, let ${\tilde{C}}_{t}$ denote the averaged posterior median number of new infections at time *t*, and $C_{t}^{\text{true}}$ denote the corresponding averaged true epidemic curve, then

(14) $$\text{MAE}=\frac{1}{t_{\text{max}}}\overset{t_{\text{max}}}{\underset{t=1}{\sum }}\left|{\tilde{C}}_{t}-C_{t}^{\text{true}}\right|.$$

This metric provides a direct summary of pointwise discrepancy over time.

**Fig. 5:**
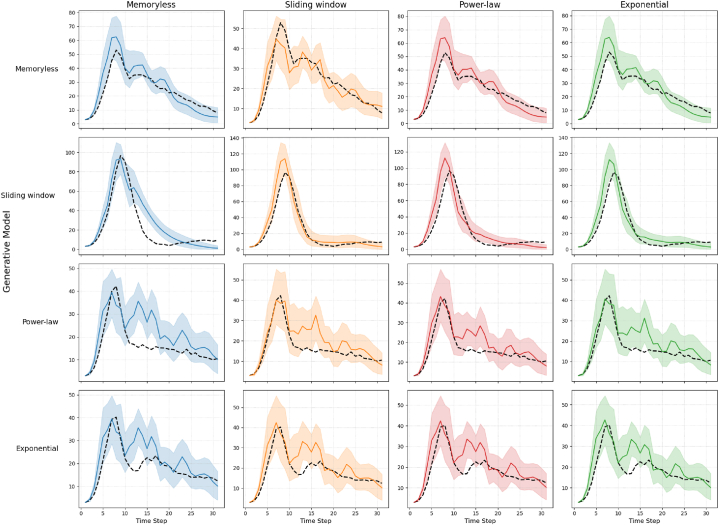
Average 95% HPDIs of epidemic curves across 20 simulated populations under each fitted model.

Fig. 5 presents the 95% HPDIs for the epidemic curves, computed from each scenario and averaged over the 20 simulated populations. The memoryless model exhibited the worst predictive performance when fitting datasets generated by the sliding window model, with its 95% HPDIs substantially exceeding the true epidemic curve during the middle time period. A similar pattern was observed when fitting datasets generated under the power-law decay memory model. In contrast, the memoryless model performed relatively better when applied to data generated from the exponential decay memory model. These differences reflect the inherent characteristics of the memory mechanisms. The memoryless model constructs its perceived risk signal using only the most recent prevalence, whereas the sliding window model incorporates information from the entire past week, and the power-law model assigns non-negligible importance to prevalence over earlier time points. Ignoring these longer-term memory effects leads to a substantial decline in predictive accuracy for the memoryless model. However, when fitting data generated by the exponential decay memory model, the discrepancy is smaller because the influence of historical data declines rapidly under this specification, reducing the mismatch.

The three models that incorporate memory effects demonstrated relatively good predictive performance when fitting datasets generated by either the memoryless mechanism or any of the memory-based mechanisms. In most scenarios, the 95% HPDIs successfully captured the true epidemic curve. These findings suggest that when the underlying memory mechanism is unknown, including a memory component in the model is more critical than selecting a specific memory form. While the traditional BC-ILM performs well when the true data-generating process is memoryless, its predictive accuracy deteriorates substantially when memory-related behaviour is present in the data. In contrast, the three MEBC-ILMs exhibit greater robustness to model misspecification, implying that using any of them would generally provide reasonable predictive performance, even in the absence of perfect knowledge of the true memory structure.

Table 2 further compares model fit using WAIC. Each row reports the average difference in WAIC between each fitted model and the benchmark true model, computed across 20 populations. All differences are positive, indicating that in every case the true model achieved better fit. Larger differences imply that the fitted model performed substantially worse relative to the true model. Consistent with the visual evidence from Fig. 5, the memoryless BC-ILM exhibited the worst fit when applied to data generated under the sliding window mechanism, while its performance was relatively better for data generated from the exponential memory model. The other three MEBC-ILMs generally showed robust model fit across most scenarios.

**Table 2:** Average WAIC difference between misspecified models and true model fits, computed across 20 populations.

	Model fit			
True model	Memoryless	Sliding window	Power-law	Exponential
Memoryless	**−−**	0.165	2.248	1.692
Sliding window	49.028	**−−**	10.972	2.449
Power-law	7.579	3.778	**−−**	2.175
Exponential	0.023	1.122	0.177	**−−**

Table 3 reports the corresponding MAE values. The MAE results are broadly consistent with both the PPD and WAIC comparisons. In particular, when the true mechanism is sliding window or power-law, the fitted memoryless model yields substantially larger errors than the memory-based alternatives. When the true mechanism is exponential, the differences among fitted models are smaller, again indicating that the memoryless model is less severely misspecified in that setting. Overall, the WAIC and MAE results reinforce the same conclusion: when behavioural memory is truly present in the data and historical prevalence meaningfully influences behavioural responses, relying on a basic BC-ILM without a memory component is not a suitable modelling choice.

**Table 3:** Mean absolute error between the averaged posterior median epidemic curve and the averaged true epidemic curve under each misspecification scenario.

	MAE			
True model	Memoryless	Sliding window	Power-law	Exponential
Memoryless	5.635	3.430	5.659	5.646
Sliding window	9.968	5.893	9.052	7.723
Power-law	6.110	4.235	3.872	4.081
Exponential	4.353	4.122	4.216	4.165

As an additional simulation, we evaluated the four MEBC-ILMs by fitting them to simulated data generated from a baseline ILM without behavioural change. The memoryless model achieved good convergence, as it could approximate the absence of behavioural change by shrinking the scale parameter *δ* towards zero. In contrast, the other three memory-based models exhibited poor convergence: although they could also reduce *δ*, the memory parameters in their alarm functions became weakly identifiable in the absence of behavioural change, hindering efficient estimation. These results highlight that when behavioural change is absent, additional flexibility introduced by memory mechanisms can reduce model identifiability and complicate inference.

## 5 Application

In this section, we apply our MEBC-ILMs in the context of the 2001 U.K. FMD epidemic. We first analyse empirical outbreak data from a subregion of Cumbria to examine how farmers adjusted their behaviour in response to reported epidemic information. We then perform a simulation study using the actual farm locations and the recorded numbers of cattle and sheep for each farm in Cumbria to evaluate whether our framework can recover the true underlying behavioural change mechanism and whether the WAIC can successfully identify it.

### 5.1 Empirical case study: 2001 U.K. FMD epidemic

We first illustrate the application of our MEBC-ILMs to empirical data from the 2001 U.K. FMD epidemic. During the epidemic, many farmers obtained timely updates on the evolving situation through television newscasts and other media sources (Hagar, 2010). The rising number of new cases likely heightened farmers' anxiety, prompting them to adopt intervention measures such as total bans on the movement of susceptible animals and restrictions on veterinarian access (Kitching et al., 2005). Conversely, when the reported rate of new infections began to decline, farmers’ protective behaviours may have relaxed (Ferguson, 2007). The objective of this case study is to use MEBC-ILMs to explore how farmers in the Cumbria region adjusted their behaviours in response to reported epidemic data and to assess whether their responses exhibit features consistent with particular memory mechanisms.

Our analysis draws on a dataset compiled by (Deeth & Deardon, 2013), containing records for 1,177 cattle and sheep farms located in a subregion of Cumbria in north-west England. The dataset provides Cartesian coordinates for each farm, along with infection and culling dates and counts of sheep and cattle present on each farm. Following Ward et al. (2025), we restricted our analysis to infection data from days 30 to 50 of the epidemic (8–28 March 2001) and fit our models to this subset of the data. Thus, this dataset is taken after animal movement bans had been introduced in February (Anderson, 2002) and any behavioural change detected would be as a result of farmers' behaviour or other government-mandated action.

To account for potential heterogeneity in farm-level susceptibility, we introduced a binary covariate *z*_*i*_ distinguishing farms by size. Following the criteria in (Deeth & Deardon, 2013), farms with more than 50 cattle or more than 200 sheep were designated as having elevated susceptibility. Under this classification, 442 farms fell into the low-susceptibility group (*z*_*i*_ = 0), while 735 farms were categorized as high-susceptibility (*z*_*i*_ = 1).

Our analysis adopts a SEIR compartmental framework to represent the transmission dynamics of the outbreak. In contrast to the SIR model outlined in Section 2, this framework introduces an additional exposed (*E*) compartment. After exposure, infected susceptible individuals enter this exposed state, meaning they are infected but not yet capable of transmitting the disease. We have set this exposed period in our work to a known and fixed duration of 5 days. The infectious period was set to a default duration of four days, except in cases where farm culling occurred earlier.

Under the BC-ILM framework, the probability of infection *P*(*i*, *t*) is formulated as

(15) $$P\left(i,t\right)=1-\exp\left[-\left(\alpha_{0}+\alpha_{1}z_{i}\right)\left(1-a_{t}\right)\underset{j\in I\left(t\right)}{\sum }{\left(d_{ij}+1\right)}^{-\beta }\right],$$

where *α*_0_ and *α*_1_ together characterize the baseline susceptibility for farms of different sizes. The time series *h*_*t*_ used to construct the alarm function *a*_*t*_, consisting of nationwide culling incidence data (i.e., the number of culled farms per day) from the entire United Kingdom, is displayed in the Supplementary Material. To characterize how local farmers may have processed the epidemic information *h*_*t*_, we interpret their behavioural responses using the four memory mechanisms introduced in Section 2.3.

Within the SEIR framework, we define the likelihood at time *t* as the product of the probabilities of infection events and non-infection events occurring at that time point:

(16) $$f_{t}\left(S\left(t\right),E\left(t\right),I\left(t\right),R\left(t\right)|\Theta \right)=\left[\underset{i\in E\left(t+1\right)\setminus E\left(t\right)}{\prod }P\left(i,t\right)\right]\left[\underset{i\in S\left(t+1\right)}{\prod }\left(1-P\left(i,t\right)\right)\right],$$

where *E*(*t*) denotes the set of exposed farms at time *t*. The full likelihood of the epidemic data *D* can therefore be expressed as

(17) $$f\left(D|\Theta \right)=\overset{t_{\text{max}}-1}{\underset{t=1}{\prod }}f_{t}\left(S\left(t\right),E\left(t\right),I\left(t\right),R\left(t\right)|\Theta \right).$$

Based on this likelihood function, we used a random walk Metropolis–Hastings MCMC algorithm to fit all four MEBC-ILM variants. For each model, we ran three independent chains of 50,000 iterations each, discarding the first 10,000 iterations as burn-in.

The Gelman–Rubin–Brooks diagnostic indicated that all four models achieved convergence. The posterior median estimates, their corresponding 95% credible intervals, and the WAIC for each model are summarized in Table 4. For the susceptibility parameters *α*_0_ and *α*_1_, as well as the transmissibility parameter *β*, all four models produced broadly similar estimates. The primary differences among the models were observed in the parameters associated with the alarm function. In particular, the scale parameter *δ* is not directly comparable across models because it scales model-specific risk signals *ψ*_*t*_ with different units and magnitudes. The scale parameter *δ* in the memoryless and sliding window models tended to have larger estimated values, reflecting the nature of their respective risk signals *ψ*_*t*_. Because these two models incorporate less historical information when constructing *ψ*_*t*_, the scale of their risk signal is generally smaller than that in the power-law and exponential memory models, which effectively summarize information from day 0 onward. As a result, larger values of *δ* are required in the memoryless and sliding window models to produce comparable alarm function values. The median estimate for the forgetting rate in the power-law memory model was 1.1678, while that for the exponential memory model was 1.0524. The power-law memory model exhibits slower decay. Based on the posterior median estimate, the 3-day decay level is approximately *D*_3_ = 0.28, and the contribution of information from 14 days earlier remains around *D*_14_ = 0.05. In contrast, the exponential memory model decays more rapidly, with *D*_3_ = 0.08 and the contribution from earlier observations becoming negligible by around day 6. Because the power-law model retains historical information more strongly, the resulting *ψ*_*t*_ values tend to be larger on average, which in turn leads to smaller posterior estimates of the scale parameter *δ*.

**Table 4:** Posterior median estimates, 95% credible intervals, and WAIC for each model.

Basic BC-ILM (Memoryless model)				
Parameter	2.5% CI	97.5% CI	Median Estimate	WAIC
*α*_0_	0.0087	0.0432	0.0216	1576.33
*α*_1_	0.0456	0.1778	0.0976	
*β*	1.7671	2.3530	2.0655	
*δ*	0.0024	0.0188	0.0103	

Sliding window memory model				
Parameter	2.5% CI	97.5% CI	Median Estimate	WAIC
*α*_0_	0.0099	0.0517	0.0253	1573.61
*α*_1_	0.0527	0.2082	0.1135	
*β*	1.7698	2.3473	2.0672	
*δ*	0.0044	0.0260	0.0150	
*k*_max_	1	7	5	

Power-law decay memory model				
Parameter	2.5% CI	97.5% CI	Median Estimate	WAIC
*α*_0_	0.0092	0.0458	0.0234	1574.63
*α*_1_	0.0477	0.1962	0.1051	
*β*	1.7752	2.3658	2.0674	
*δ*	0.0007	0.0109	0.0043	
*λ*_*P*_	0.0653	3.6290	1.1678	

Exponential decay memory model				
Parameter	2.5% CI	97.5% CI	Median Estimate	WAIC
*α*_0_	0.0095	0.0456	0.0227	1574.94
*α*_1_	0.0498	0.1795	0.1014	
*β*	1.7817	2.3399	2.0588	
*δ*	0.0012	0.0127	0.0057	
*λ*_*E*_	0.0452	3.9256	1.0524	

**Fig. 6:**
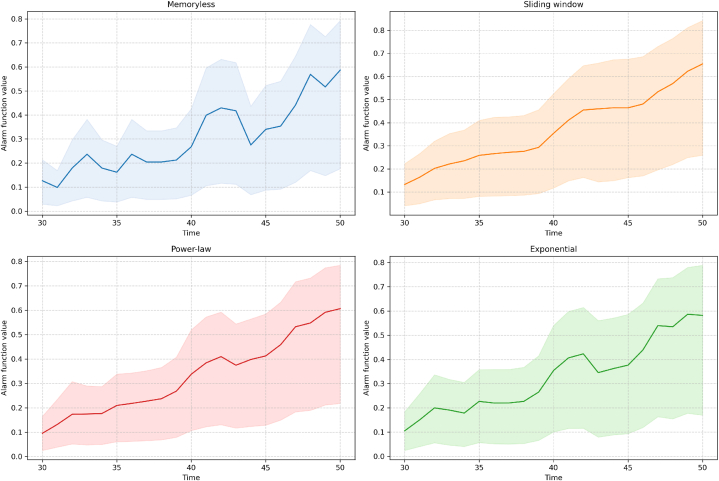
Estimated daily alarm function values and 95% HPDIs under four MEBC-ILMs.

The four models yielded similar WAIC values, with the sliding window model performing slightly better than the others. This suggests that all models provide a reasonable explanation of the behavioural change observed in the data. Fig. 6 presents the estimated daily alarm function values and their associated 95% HPDIs for all four models. Overall, the trends are broadly similar across models, with alarm levels intensifying as the epidemic progressed. Among them, the sliding window model exhibited the smoothest increase in alarm values, consistent with its functional form: because the alarm at each time point reflects the average culling incidence over several previous days, sudden spikes or drops in daily counts have a dampened impact on the perceived risk. In our formulation, we assume that farmers actively monitor epidemic data on a daily basis. However, in practice, occasional lapses in attention may occur, with individuals basing their risk assessments on information from prior days. In such contexts, the robustness of the sliding window function becomes particularly valuable. In contrast, the memoryless model demonstrated more variability, with alarm values showing pronounced peaks and troughs that closely track daily fluctuations in epidemic data. This behaviour reflects the model's sensitivity to the most recent information, making it particularly suited for capturing real-time responses among individuals highly attentive to current developments. The exponential model behaved similarly to the memoryless model, as its fast forgetting rate causes earlier historical data to have little influence on perceived risk, thereby emphasizing recent observations. The power-law model, by comparison, decays more slowly, allowing past information to retain a non-negligible influence over a longer time horizon, and consequently producing more stable alarm trajectories. These differences reflect the structural assumptions built into each model and highlight that, although the four MEBC-ILMs differ in how they incorporate historical information into the perceived risk signal, they are all capable of capturing the behavioural change effects present in the data.

### 5.2 Simulation study in the FMD context

The empirical analysis in Section 5.1 did not reveal a clear preference for any particular memory mechanism. To further investigate, we carried out a simulation study to see whether our framework can recover the true behavioural change mechanism when it is present. We used the actual farm locations and the recorded numbers of cattle and sheep from the Cumbria dataset to generate 20 simulated epidemics under the sliding window MEBC-ILM, taken here as the true generative model. The true parameter values were *α*_0_ = 0.1, *α*_1_ = 0.4, *β* = 2, *δ* = 0.01, and *k*_max_ = 7. The latent period was fixed at 5 days and the infectious period at 4 days, with each epidemic simulated over 20 days.

In these simulations, farmers were assumed to form their perceived risk based on the prevalence, defined as the number of currently infected farms at each time step. In each dataset, three farms were randomly chosen from the 1,177 farms to serve as the initial infected premises. For each of the 20 datasets, we fitted all four MEBC-ILM variants and calculated the WAIC for each fit to check whether WAIC can identify the true model in the spatial and temporal context of the FMD outbreak.

The Gelman–Rubin–Brooks diagnostic showed that the memoryless model and the sliding window model converged in all simulated datasets. The power-law and exponential models converged much less often, with the power-law model converging for 8 datasets and the exponential model for only 2. WAIC values were therefore not computed for these two models. Of course, the poor convergence of those two models suggests a poor fit to the data generated under the sliding-window model.

**Fig. 7:**
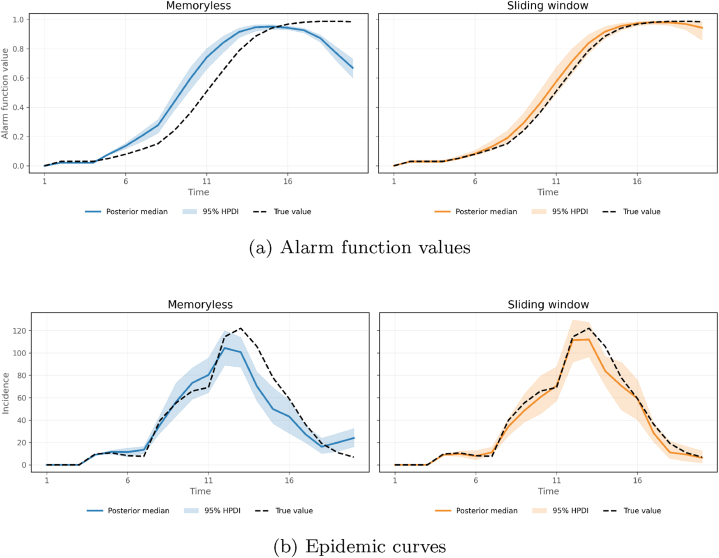
Comparison of 95% HPDIs for alarm function values and daily incidence between the memoryless and sliding window models, averaged over 20 simulations, with true values shown for reference.

Fig. 7 shows the 95% HPDIs for daily incidence and alarm function values, averaged over 20 simulations. The sliding window model has strong predictive ability, with its 95% HPDI almost always covering the true values. The memoryless model performs poorly for the alarm function: as the epidemic progresses and daily prevalence declines, it assumes the alarm function value should also drop, which leads to large deviations from the truth. Its epidemic curve predictions also show more bias, even forecasting a resurgence at the end that is far from the observed trend. When a memory mechanism is present, using a memoryless model is therefore not a sound choice.

The WAIC results in the Supplementary Material match the visual impression from the figure. The sliding window model consistently produces lower WAIC values, on average about 47 points below the memoryless model. This suggests that when a memory mechanism exists, WAIC can help identify the model that better reflects the underlying transmission dynamics.

## 6 Discussion and conclusion

Understanding how individuals adjust their behaviours in response to evolving epidemic conditions is critical for accurate modelling and intervention planning. This study developed a flexible framework of data-driven MEBC-ILMs within a Bayesian statistical framework to better capture how individuals may process epidemic information over time when adjusting their behaviours. By extending existing behavioural change epidemic models, we introduced four distinct specifications for modelling memory, each reflecting different assumptions about how historical epidemiological data contribute to perceived risk. The proposed framework allows behavioural responses to be dynamically modulated by past epidemic trends and provides a unified structure for comparing competing memory assumptions. Through a series of simulation studies and an application to data from the 2001 U.K. FMD epidemic, we demonstrated that these models are capable of capturing behavioural change effects under a range of settings, while also offering insights into the underlying temporal structure of information use in behavioural responses. Our analysis further showed that MEBC-ILMs exhibit considerable robustness to model misspecification: when used to fit data generated from different memory processes or from a memoryless setting, their predictive performance generally remained adequate. By contrast, when the true behavioural process incorporated memory but was analysed using the basic BC-ILM, predictive performance deteriorated substantially. This underscores the value of explicitly accounting for memory structures when modelling behavioural responses to epidemics.

Our future research can focus on several directions. Firstly, our model assumes homogeneity in population-level behaviour change. However, in reality, individuals often process information differently. People residing in areas severely affected by the epidemic might monitor daily infection data more closely, leading to a profound memory of recent historical data. Conversely, those in relatively safer communities might be more relaxed, with a less acute recollection of epidemic figures. Other demographic characteristics, such as age and education level, could also influence individuals' attention to epidemic data, thereby affecting their subsequent behavioural changes (Paasche-Orlow & Wolf, 2007; Tennant et al., 2015). Incorporating these covariates into future models may enable more effective heterogeneity analysis, thus enhancing the model's predictive capability.

Secondly, our model assumes that individuals form risk perception based on a single time series, such as daily prevalence data. While this assumption might be reasonable in a media-undeveloped setting, the current era is characterized by an information explosion, where people can easily access vast amounts of epidemic-related data online. For instance, during the COVID-19 pandemic, individuals could readily obtain data on mortality rates, ICU occupancy, positivity rates, and other metrics from platforms like the CDC COVID Data Tracker (Centers for Disease Control and Prevention, 2025). Moving forward, research could attempt to incorporate multiple information sources beyond a single time series, assigning different weights of importance to each source to more accurately describe the formation of perceived risk. Alternatively, given the sheer volume of data accessible to the public, including all of it in the model is clearly not a practical solution. Therefore, related decision science research, such as using discrete choice experiments (Mao et al., 2025; Train, 2009) to determine the relative importance of these data in shaping public risk perception, would offer significant guidance for our modelling efforts.

Thirdly, as the first attempt to incorporate memory mechanisms into the ILM framework, our work lays the foundation for future extensions to more complex ILMs. These include multistrain ILMs, which model the simultaneous spread of multiple interacting pathogen strains within the system (Romanescu & Deardon, 2016); continuous-time ILMs, which model transmission dynamics in continuous time rather than discrete intervals (Almutiry et al., 2021); network-based ILMs, which represent transmission through explicit or latent contact networks (Almutiry & Deardon, 2021); geographically dependent ILMs, which allow transmission probabilities to depend directly on spatial location and accommodate the effects of spatially varying risk factors (Amiri et al., 2023, 2024; Mahsin et al., 2022); non-parametric ILMs, which avoid restrictive parametric assumptions and flexibly capture complex spatial or transmission structures (Rahul & Deardon, 2024, 2025); and directional spatial ILMs, which incorporate directional transmission tendencies such as movement patterns (Peitsch et al., 2026).

Finally, our current modelling approach maintains analytical simplicity and manageable computational demands by operating under the assumption of fixed and predetermined latent and infectious periods, together with infection histories that are effectively fully observed. The conclusions drawn from this study should therefore be interpreted primarily as a methodological comparison under idealized observation, rather than as a direct framework for partially observed surveillance data. In practice, these assumptions often diverge from real-world settings, where crucial epidemiological timelines may vary substantially across individuals, and where the precise timing of events such as exposure, the onset of infectiousness, and removal from the infectious pool is frequently unobserved or censored. To account for such uncertainties, future research could incorporate more flexible inferential approaches, including data-augmented MCMC techniques, although this would entail a considerable computational burden. Recent work has proposed approximate inference methods that may help reduce this burden in such settings (Deeth & Deardon, 2016; Liu et al., 2021; Pokharel & Deardon, 2022; Ward et al., 2022).

## CRediT authorship contribution statement

**Yicheng Mao:** Writing – review & editing, Writing – original draft, Visualization, Validation, Software, Methodology, Investigation, Formal analysis, Conceptualization. **Rob Deardon:** Writing – review & editing, Supervision, Methodology, Conceptualization. **Lorna E. Deeth:** Writing – review & editing, Supervision.
