# Supplementary material for: Memory mechanisms for behavioural change in Bayesian individual-level spatial epidemic models
Source: Infect Dis Model. 2026 Jun 2;11(4):1536–53. doi: 10.1016/j.idm.2026.05.008 (PMC13276345; doi:10.1016/j.idm.2026.05.008)
Supplement: Multimedia component 1 [file mmc1.pdf]

Supplementary Material to Memory Mechanisms for Behavioural Change in Bayesian Individual-Level Spatial Epidemic Models

Yicheng Mao[1,2,*], Rob Deardon[2,3], and Lorna E. Deeth[4]

[1]Department of Data Analytics and Digitalization, Maastricht University, P.O. Box 616, 6200 MD Maastricht, The Netherlands
[2]Department of Mathematics and Statistics, University of Calgary, University Drive NW, Calgary, T2N 1N4, Canada
[3]Faculty of Veterinary Medicine, University of Calgary, University Drive NW, Calgary, T2N 1N4, Canada
[4]Department of Mathematics and Statistics, University of Guelph, 50 Stone Rd. E., Guelph, Ontario N1G 2W1, Canada
[*]Correspondence: yicheng.mao1@ucalgary.ca

S1 Supplementary Analysis

S1.1 Alternative Behavioural Formulations and Resulting Epidemic Patterns

In the main text, behavioural change is incorporated through a susceptibility-scaling formulation, in which the alarm function reduces infection risk by modifying the susceptibility term. Ward et al. (2025) also discussed an alternative specification in which behavioural response acts more directly through the transmission kernel, so that alarm affects the effective spatial interaction structure. We write this alternative formulation as

$$P(i,t) = 1 - \exp\left[-\alpha \sum_{j \in I(t)} (d_{ij}+1)^{-\beta(1-a_t)^{-1}}\right]. \tag{S1}$$

Here, one is added to the Euclidean distance to keep the modified kernel well behaved when individuals are in very close proximity. This avoids the counterintuitive situation in which stronger behavioural effects would otherwise increase transmission at very small distances. For convenience, we refer to the formulation used in the main text as susceptibility-based scaling, and to Eq. (S1) as kernel-based scaling.

In addition, the exponential alarm function used in the main text is not the only alarm specification considered in earlier work. Both Ward et al. (2023) and Ward et al. (2025) discussed several alternative alarm functions that differ in how perceived risk is translated into behavioural response. Table S1 summarizes three such alternatives.

To examine how epidemic dynamics change across these behavioural specifications, we simulated epidemics under all combinations of alarm type, response structure, and alarm intensity. In all simulations, the perceived risk signal was constructed using the power-law memory model. We considered two memory settings, $\lambda_P = 1.0$ and

Table S1: Alternative alarm function specifications discussed by Ward et al. (2023) and Ward et al. (2025).

Alarm type	Functional form	Description
Threshold	$a_t = \begin{cases} \delta_1, & \text{if } \psi_t > \delta_2, \\ 0, & \text{otherwise} \end{cases}$	A piecewise response that remains inactive below a risk threshold and shifts abruptly to a fixed alarm level once that threshold is crossed.
Power	$a_t = 1 - (1 - \psi_t)^{1/\delta}$	A smooth nonlinear response in which the alarm level increases more rapidly as perceived risk becomes larger.
Hill-type	$a_t = \dfrac{\psi_t^{\delta_2}}{\delta_1^{\delta_2} + \psi_t^{\delta_2}}$	A saturating response in which δ_1 governs the midpoint of the transition and δ_2 determines how sharply the alarm level rises around that point.

$\lambda_P = 1.8$, corresponding to relatively long and short memory, respectively. For each combination, we generated 20 independent populations of size $N = 1{,}000$, with spatial locations drawn independently from a uniform distribution over a 200×200 square. Five individuals were randomly selected as the initial infectious cases, the infectious period was fixed at 3 days, and the epidemic was simulated for 30 discrete time steps under the SIR framework. To keep the comparison focused on behavioural effects, the spatial decay parameter was fixed at $\beta = 2.0$, while α was set to 3.0 under susceptibility-based scaling and 5.0 under kernel-based scaling to place the two formulations on broadly comparable epidemic scales. Within each alarm type and response structure, we considered three alarm intensities, labelled weak, moderate, and strong. The full parameter settings are reported in Table S2.

Figures S1 and S2 show the resulting mean incidence curves, averaged across the 20 simulated populations, with shaded bands indicating the corresponding 95% intervals. Across nearly all settings, increasing alarm intensity lowers the epidemic peak and reduces cumulative incidence, consistent with the interpretation that stronger behavioural response more effectively suppresses transmission. This pattern is clearest under the exponential and power alarms, where weak, moderate, and strong settings generate a fairly ordered progression in epidemic size. The threshold and Hill-type alarms also show strong suppression effects, although the change across levels is sometimes less smooth because these response functions introduce sharper nonlinearities into the behavioural feedback.

The two structural formulations differ substantially in how strongly alarm suppresses transmission. Under susceptibility-based scaling, the alarm enters linearly through the factor $(1 - a_t)$, so increasing alarm strength dampens transmission in a relatively direct way. Under kernel-based scaling, by contrast, alarm enters through the exponent of the distance term, which makes transmission more sensitive to behavioural response. As a result, the kernel-based formulation often produces stronger suppression than the susceptibility-based formulation, particularly under the exponential and power alarms. This contrast is less pronounced for the threshold alarm, where the binary on-off structure of the alarm dominates the response once activation occurs.

The memory parameter also plays an important role in shaping epidemic trajectories. Under long memory, past

Table S2: Simulation parameter settings for the alternative behavioural formulations.

			Susceptibility-based				Kernel-based			
λ_P	Alarm type	Level	α	β	δ_1	δ_2	α	β	δ_1	δ_2
Short memory ($\lambda_P = 1.8$)	Exponential	Weak	3.0	2.0	0.005	—	5.0	2.0	0.002	—
		Moderate	3.0	2.0	0.012	—	5.0	2.0	0.004	—
		Strong	3.0	2.0	0.025	—	5.0	2.0	0.008	—
	Power	Weak	3.0	2.0	0.15	—	5.0	2.0	0.15	—
		Moderate	3.0	2.0	0.08	—	5.0	2.0	0.08	—
		Strong	3.0	2.0	0.04	—	5.0	2.0	0.04	—
	Threshold	Weak	3.0	2.0	0.30	15	5.0	2.0	0.20	15
		Moderate	3.0	2.0	0.50	15	5.0	2.0	0.40	15
		Strong	3.0	2.0	0.70	15	5.0	2.0	0.60	15
	Hill	Weak	3.0	2.0	0.10	3	5.0	2.0	0.10	3
		Moderate	3.0	2.0	0.06	3	5.0	2.0	0.06	3
		Strong	3.0	2.0	0.04	3	5.0	2.0	0.04	3
Long memory ($\lambda_P = 1.0$)	Exponential	Weak	3.0	2.0	0.005	—	5.0	2.0	0.002	—
		Moderate	3.0	2.0	0.012	—	5.0	2.0	0.004	—
		Strong	3.0	2.0	0.025	—	5.0	2.0	0.008	—
	Power	Weak	3.0	2.0	0.15	—	5.0	2.0	0.15	—
		Moderate	3.0	2.0	0.08	—	5.0	2.0	0.08	—
		Strong	3.0	2.0	0.04	—	5.0	2.0	0.04	—
	Threshold	Weak	3.0	2.0	0.30	15	5.0	2.0	0.20	15
		Moderate	3.0	2.0	0.50	15	5.0	2.0	0.40	15
		Strong	3.0	2.0	0.70	15	5.0	2.0	0.60	15
	Hill	Weak	3.0	2.0	0.10	3	5.0	2.0	0.10	3
		Moderate	3.0	2.0	0.06	3	5.0	2.0	0.06	3
		Strong	3.0	2.0	0.04	3	5.0	2.0	0.04	3

incidence continues to influence the perceived risk signal for a longer period, which tends to sustain behavioural response and suppress later transmission. This effect is most evident for the exponential, power, and Hill-type alarms, where the long-memory panels generally show lower late-stage incidence and weaker rebounds than the short-memory panels. By contrast, the threshold alarm is much less sensitive to the distinction between short and long memory, because its activation depends primarily on whether the perceived risk signal crosses a fixed threshold rather than on gradual variation in alarm magnitude.

The supplementary simulations also show that the current modelling framework can generate oscillatory or multi-wave epidemic patterns, although such behaviour is not observed uniformly across formulations. In several settings, particularly under the exponential, power, and Hill-type alarms in the short-memory case, the simulated trajectories exhibit delayed rebounds or secondary increases in incidence after the initial peak. These patterns are more apparent under susceptibility-based scaling, where the first wave is moderated but not completely suppressed, allowing transmission to increase again as alarm weakens. However, these results do not suggest that behavioural feedback alone is sufficient to account for all multi-wave epidemics observed in practice. In more realistic settings, repeated waves may also depend on additional epidemiological mechanisms, such as waning immunity and reinfection in SIRS-

type models, as well as more complex behavioural processes, including time-varying responses and heterogeneous behavioural adaptation across the population.

S1.2 Sensitivity Analysis with Respect to Susceptible Depletion

To further investigate whether the systematic underestimation observed in the main simulation study was related to the stage of susceptible depletion, we conducted a supplementary sensitivity analysis using the power-law memory model as a representative example.

We generated 20 epidemic datasets under a spatial SIR framework with $\alpha = 2.4$, $\beta = 2$, $\delta = 0.01$, and $\lambda_P = 1$, using the same population size and spatial layout as in the main simulation study. In each dataset, three individuals were randomly selected as the initial infectious cases, and the epidemic was simulated for 30 discrete time steps. We then examined the average trajectory of the susceptible proportion across the 20 simulated populations, shown in Figure S3. Based on this trajectory, we selected three observation windows for repeated inference: the first 7 days, the first 15 days, and the full 30-day epidemic period. These three horizons approximately correspond to an early phase, an intermediate phase, and the full epidemic course, and were chosen to reflect progressively stronger susceptible depletion over time.

For each observation window, inference was conducted using three parallel MCMC chains, each with 50,000 iterations and the first 10,000 discarded as burn-in. Convergence was satisfactory in all cases, with all potential scale reduction factors below 1.05.

Figure S4 compares the posterior means and 95% credible intervals across the three observation windows for all 20 simulated populations. The three horizons show different patterns across parameters. For α, the 7-day analysis is generally closer to the true value than the 30-day analysis, with the 15-day analysis lying between them, suggesting that the later epidemic phase contributes to the downward bias in this parameter. A similar but less pronounced pattern is visible for β. For δ, the 7-day window produces wider credible intervals and somewhat greater variability across populations, while the 15-day and 30-day analyses are more concentrated. This pattern is even clearer for λ_P, for which the shortest window leads to substantially wider intervals and greater between-population variation, whereas the 15-day and 30-day analyses are more tightly centered around the true value. Overall, these results suggest that restricting inference to earlier epidemic stages can reduce some of the downward bias in the transmission-related parameters, but at the same time provides less information about the behavioural and memory parameters.

This interpretation is further supported by the posterior geometry shown in Figure S5, which presents posterior pair plots for one representative population under the 7-day, 15-day, and 30-day windows. The posterior distributions are much more diffuse under the 7-day window, especially for δ and λ_P, which is consistent with the wider intervals seen in Figure S4. As the observation window increases, the posteriors become more concentrated, but the dependence among parameters remains visible. In particular, the association between α and β persists across all

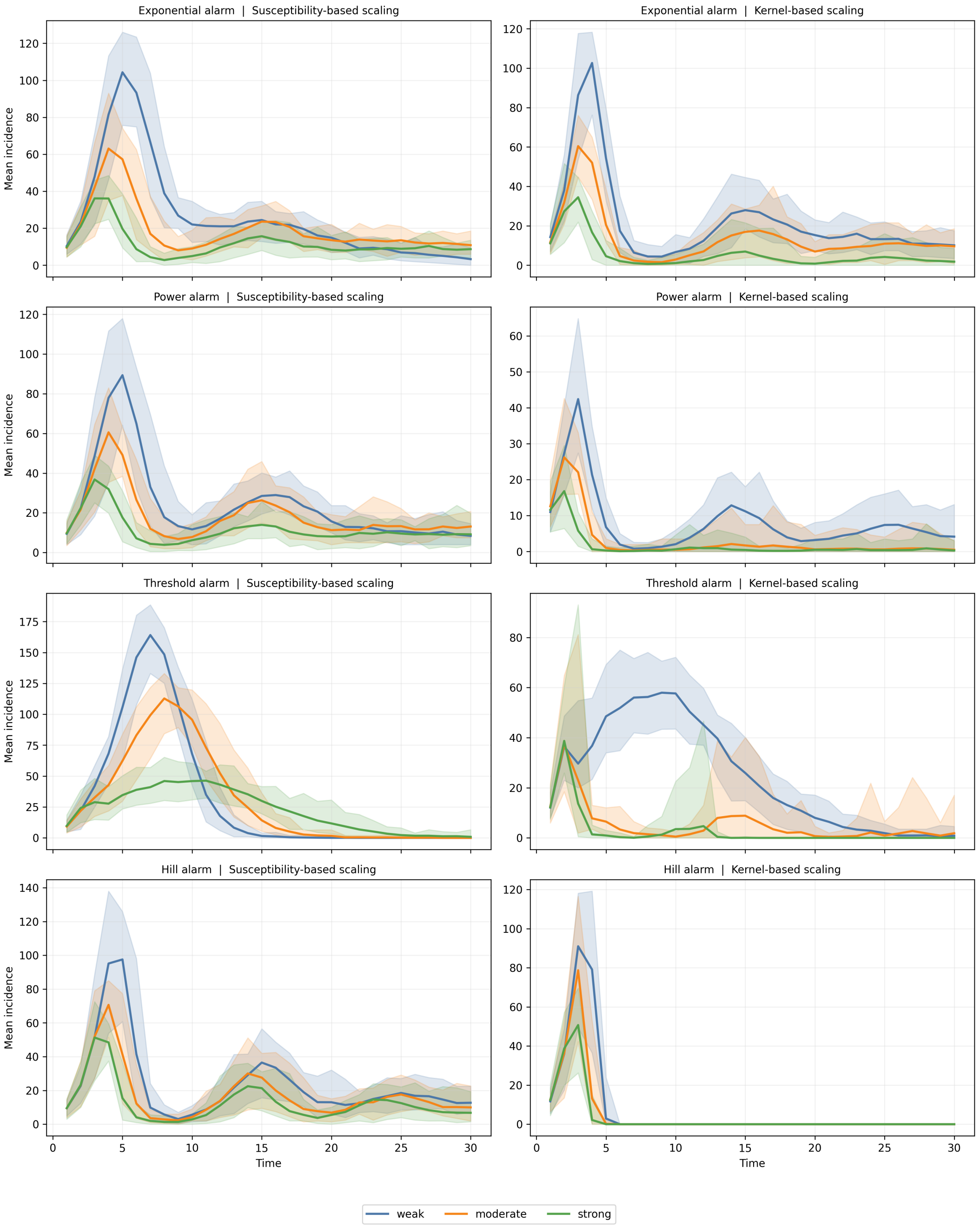

Figure S1: Mean incidence curves under the alternative behavioural formulations with short memory ($\lambda_P = 1.8$). Within each panel, the three curves correspond to weak, moderate, and strong alarm intensity settings, and the shaded regions show the corresponding 95% intervals across 20 simulated populations.

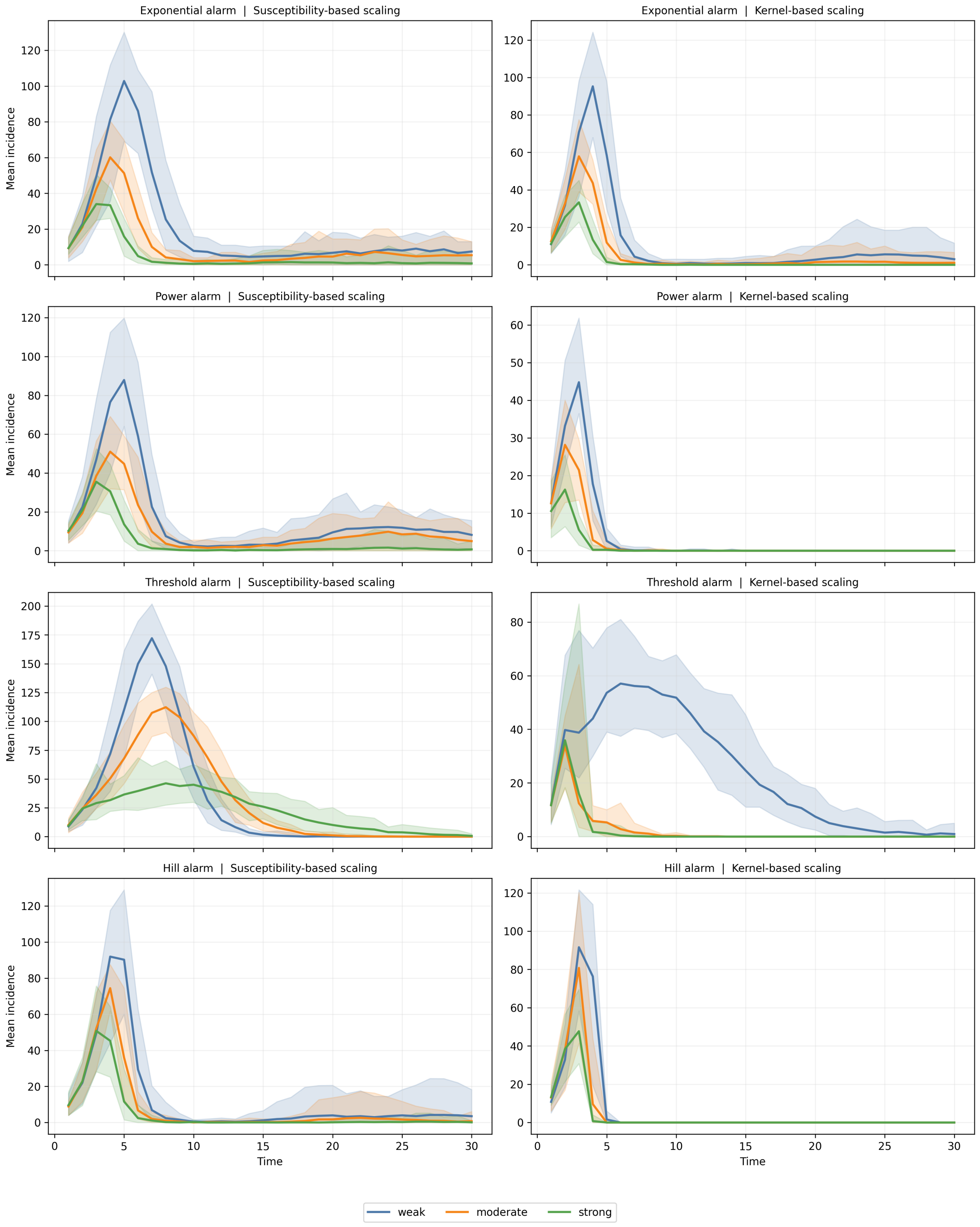

Figure S2: Mean incidence curves under the alternative behavioural formulations with long memory ($\lambda_P = 1.0$). Within each panel, the three curves correspond to weak, moderate, and strong alarm intensity settings, and the shaded regions show the corresponding 95% intervals across 20 simulated populations.

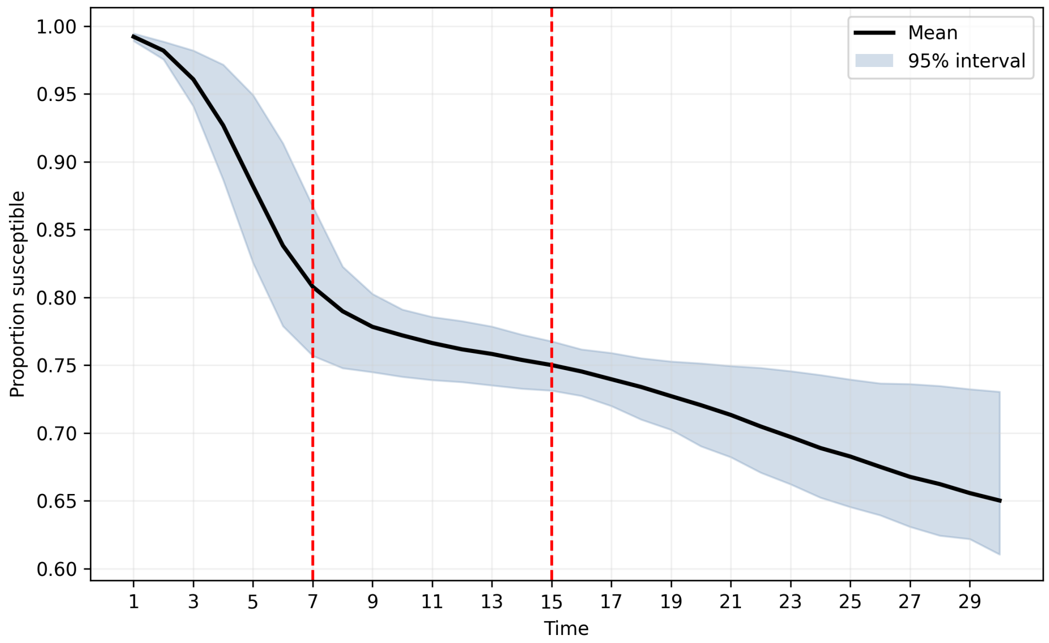

Figure S3: Average susceptible proportion across 20 simulated populations under the power-law memory model. The solid line shows the mean and the shaded region shows the 95% interval. The red dashed vertical lines at days 7 and 15 indicate the cutoffs used to define the early and intermediate inference windows.

three horizons, indicating that parameter trade-offs are present even when inference is restricted to earlier epidemic stages. Taken together, these supplementary results suggest that both susceptible depletion and dependence among model parameters are important contributors to the bias observed in the main simulation study.

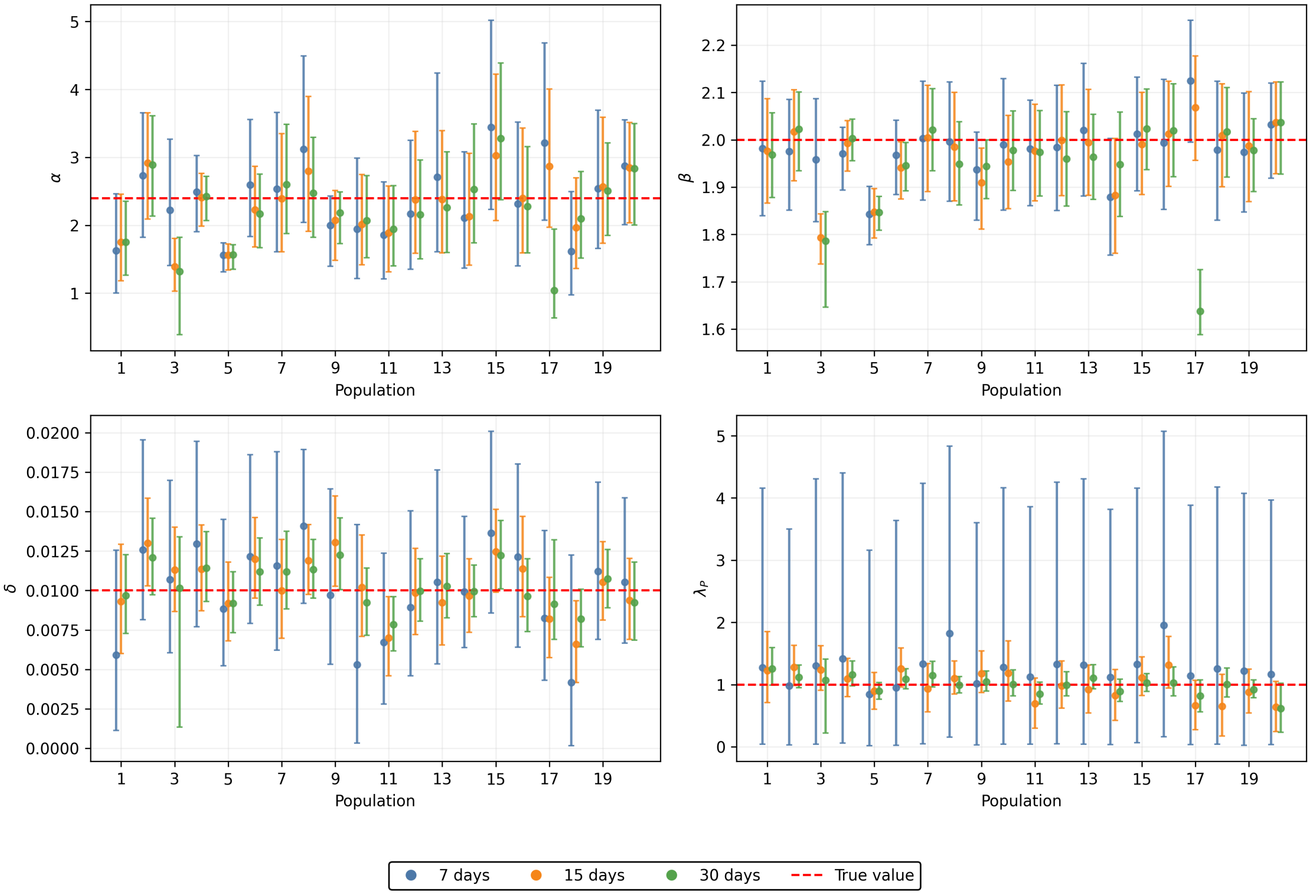

Figure S4: Posterior means and 95% credible intervals for α, β, δ, and λ_P across the 20 simulated populations under the 7-day, 15-day, and 30-day observation windows. The red dashed lines indicate the true parameter values.

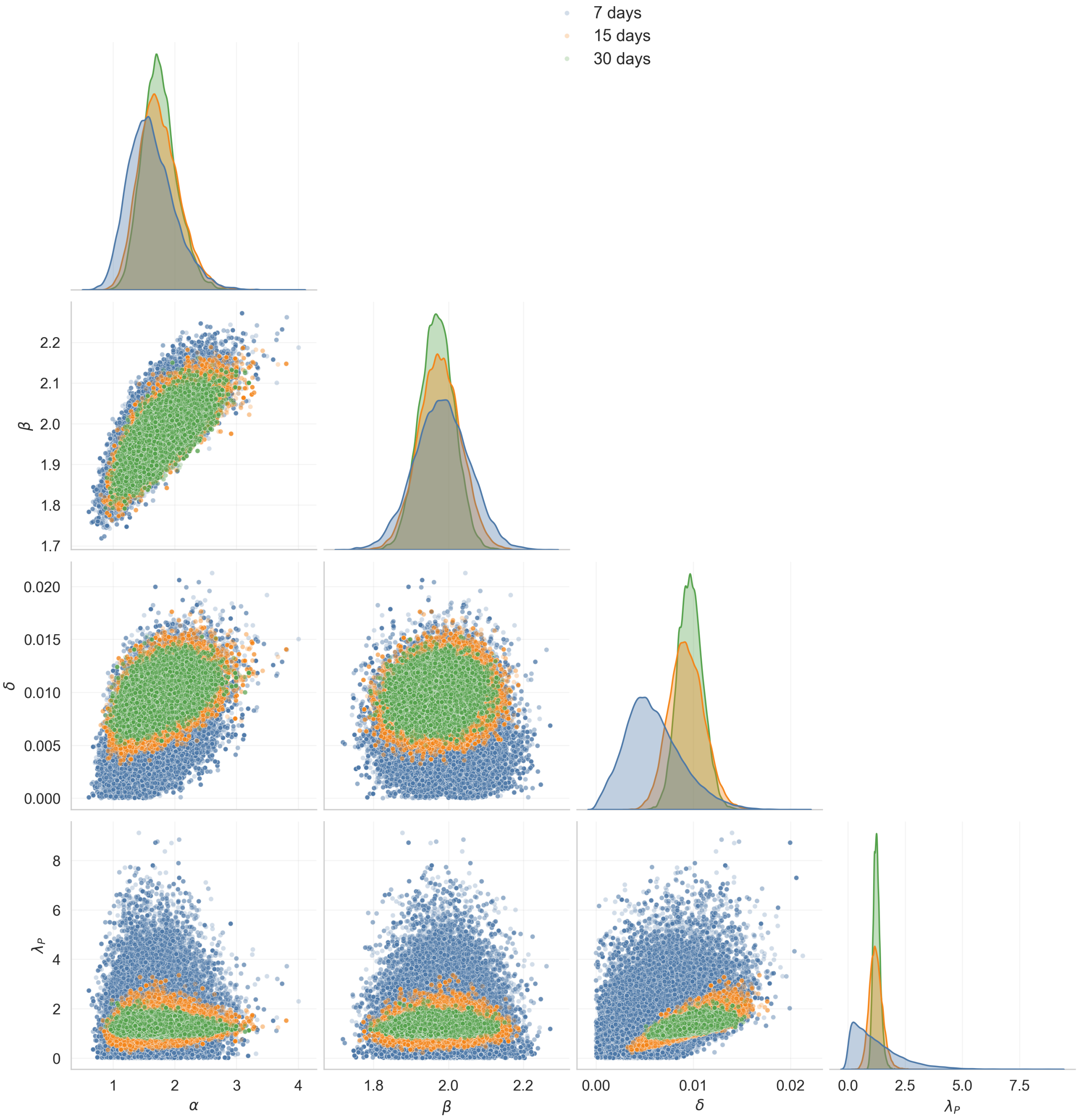

Figure S5: Posterior pair plots for one representative simulated population under the 7-day, 15-day, and 30-day observation windows. Different colours correspond to different inference horizons.

S2 Supplementary Tables and Figures

Table S3: WAIC comparison between memoryless and sliding window models over 20 simulations.

Simulation	Memoryless	Sliding window
1	5137.15	5085.58
2	5006.34	4972.20
3	4880.20	4820.41
4	4980.44	4933.76
5	4902.77	4861.64
6	4884.03	4831.79
7	5069.65	5024.49
8	4957.46	4925.94
9	5081.34	5036.40
10	5015.91	4961.30
11	4966.18	4920.60
12	5072.94	5040.16
13	4909.39	4895.27
14	5071.16	5036.08
15	4896.96	4822.40
16	5070.20	5020.97
17	5053.74	5006.14
18	4838.32	4783.04
19	4880.02	4805.63
20	4865.84	4818.65
Mean	4977.00	4930.12

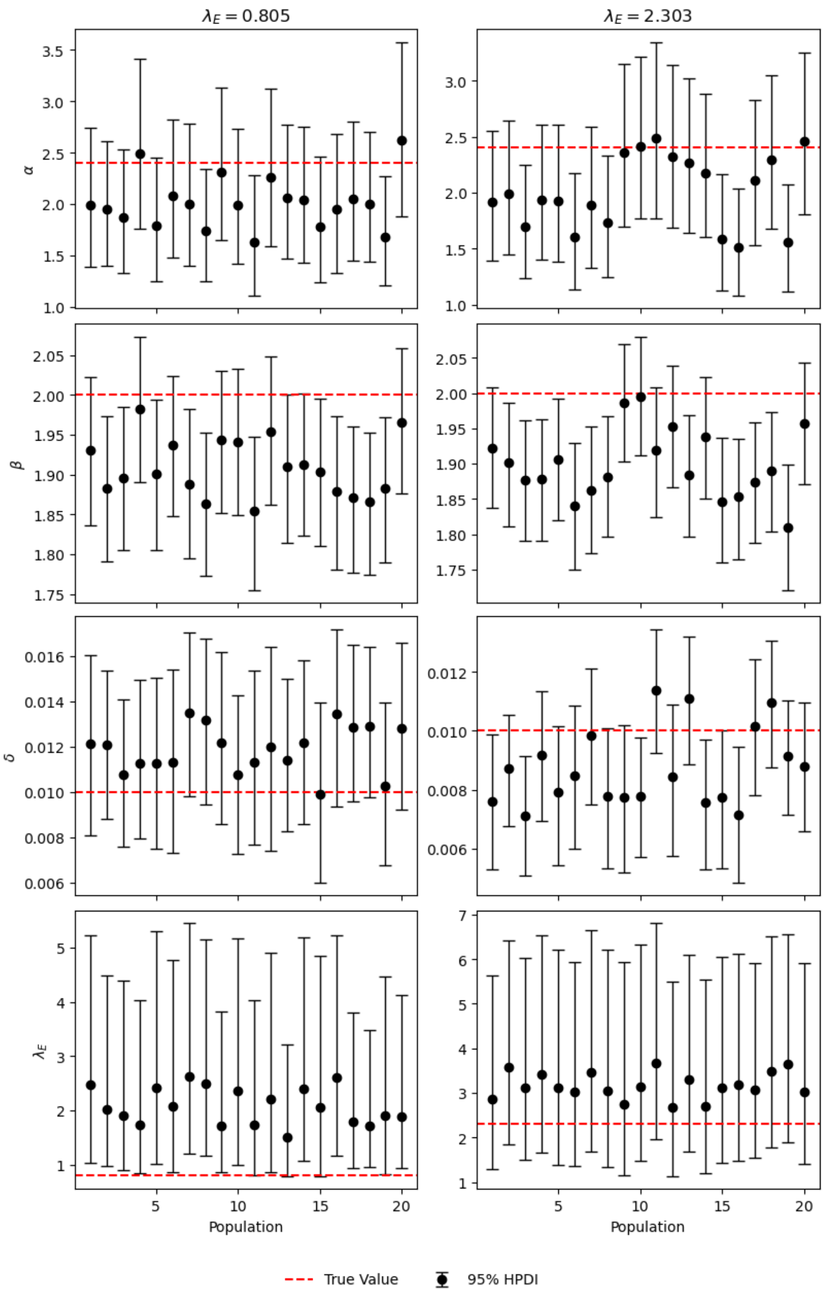

Figure S6: Posterior summaries of α, β, δ, and λ_E under the exponential memory specification.

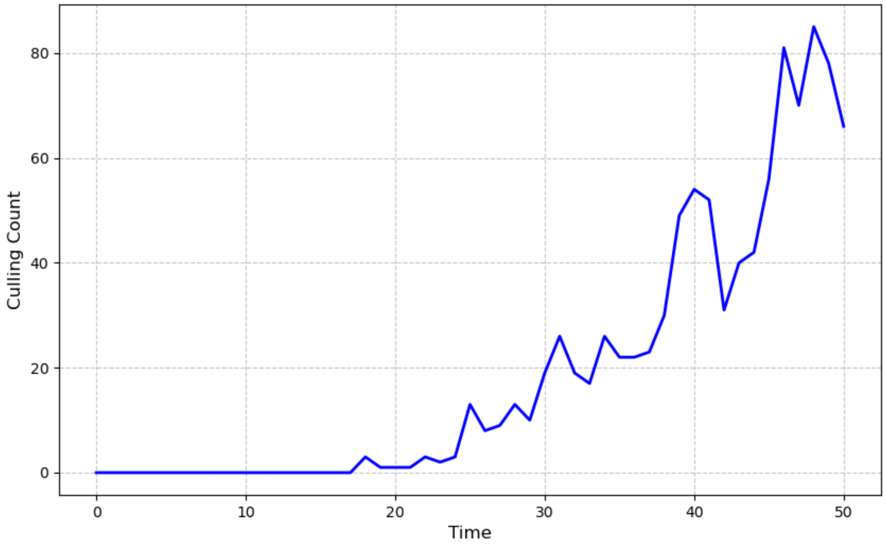

Figure S7: Temporal trends in the number of culled farms during the study period.

References

Ward, C., Deardon, R., and Schmidt, A. M. (2023). Bayesian modeling of dynamic behavioral change during an epidemic. *Infectious Disease Modelling*, 8(4):947–963.

Ward, M. A., Deardon, R., and Deeth, L. E. (2025). A framework for incorporating behavioural change into individual-level spatial epidemic models. *Canadian Journal of Statistics*, 53(1):e11828.
